# Dynamics of rhizosphere bacterial communities and soil physiochemical properties in response to consecutive ratooning of sugarcane

**DOI:** 10.3389/fmicb.2023.1197246

**Published:** 2023-07-10

**Authors:** Abdullah Khan, Yibin Wei, Muhammad Adnan, Izhar Ali, Muqing Zhang

**Affiliations:** ^1^Guangxi Key Laboratory of Sugarcane Biology, State Key Laboratory for Conservation and Utilization of Subtropical Agro-Bioresources, Guangxi University, Nanning, China; ^2^Department of Plant Protection, College of Agriculture, Guangxi University, Nanning, China; ^3^Department of Plant Sciences, The University of Agriculture Peshawar, Peshawar, Pakistan; ^4^Guangxi Key Laboratory of Forest Ecology and Conservation, College of Forestry, Guangxi University, Nanning, China

**Keywords:** sugarcane, ratooning, soil nutrients, bacterial diversity, land degradation

## Abstract

Ratooning in sugarcane often leads to soil problems such as degradation, acidification, and soil-borne diseases that negatively impact agriculture output and sustainability. Understanding the alteration in bacterial communities, activities, and their diversity connected to the plant and soil under consecutive ratooning still needs to be clarified. To address this gap, multidisciplinary approaches such as Illumina sequencing and measurement of soil nutrients and enzymes were used in this study to analyze soil samples in a field with three consecutive ratooning sugarcane crops. The results revealed a decline in crop yield and significant changes (*P* < 0.05) in soil nutrients and bacterial diversity. Ratooning resulted in an acidic environment that potentially affected soil nutrients and enzyme activity responsible for the cycling of carbon, nitrogen, and phosphorous. Non-metric dimensional scaling (NMDS) confirmed the effect of ratooning on soil attributes. Moreover, a positive correlation between soil physiochemical properties and soil enzymes was observed. Alpha diversity indices indicated greater bacterial diversity in ratooning sugarcane. Bacterial diversity varied throughout the ratooning crop, and significant (*P* < 0.05) changes in the relative abundance of specific phyla were observed. For example, the relative abundance of Proteobacteria was decreased, and Acidobacteria was increased. Furthermore, the relative abundance of bacterial phyla was strongly correlated with soil attributes (enzymes and nutrients). Additionally, ratooning results in the depletion or enrichment of important agriculture microbial genera such as *Sphingomonas, Burkholderia*, and *Acidothermus* (*P* < 0.05), respectively. In conclusion, ratooning led to soil acidification, decreased fertility, and altered microbial structure and activity. Thus, restraining soil acidity by means of liming or biofertilizers to maintain soil nutrients, enzymatic activities, and microbial structure could benefit plants and soil to help create a long-term eco-friendly sugarcane cropping system.

## Introduction

Sugarcane is an important industrial crop in southern China, where a vast area is vegetatively propagated and is primarily used as a raw material for the sugar industry (Khan et al., 2022a). Following the initial harvest of canes, 1–5 ratoons are typically used. However, numerous reports indicate that successive ratooning can result in declining yields even with normal crop management conditions (Xu et al., 2021). These annual decreases in sugarcane productivity may be attributed to modifications in the rhizosphere soil biology caused by ratooning sugarcane (Sheng et al., 2012). The challenges associated with the consecutive monoculture of ratooning sugarcane include low yields, degradation of soil quality over time, and increased pests and diseases (Wu et al., 2011). Allelochemicals generated by sugarcane residue and root exudates impact the composition of bacterial communities, negatively impacting the soil nutrient cycle and sugarcane growth (Gao et al., 2019).

Land degradation refers to a significant decline in soil quality and fertility, which can take various forms, including loss of nutrients, acidification, soil erosion, desertification, compaction, excessive salinity, and chemical pollution (Chen et al., 2012). Approximately 9% of agricultural land globally is severely degraded, with an additional 40% being moderately degraded, resulting in a 13% decrease in crop yields (Madramootoo, 2015). These issues often result from unsustainable farming practices such as deforestation, continuous monoculture, excessive use of fertilizers and pesticides, and overgrazing, which negatively affect soil physiochemical features and disrupt soil microbial composition (Tayyab et al., 2021). Consecutive monoculture can lead to growth and productivity challenges due to soil nutrient deficiencies and the build-up of plant-specific microbes that may harm crops (Chen et al., 2012). Continuous monoculture is a farming practice involving repeatedly growing the same crop on the same land to achieve high yields. However, this approach can have detrimental effects on soil quality and plant growth, ultimately leading to decreased crop production (Chi et al., 2017; Chen et al., 2018). This approach results in a decline in soil quality, characterized by several factors such as depletion of soil carbon, inadequate nutrient levels, diminished diversity, lowered land productivity, soil acidification, salinity, compactness, and modifications to the soil microbial population (Kepler et al., 2012; Arafat et al., 2017). Continuous ratooning, a form of monoculture, is known to stunt plant growth, decrease plant vigor, and result in lower crop production, posing a threat to agricultural areas (Chen et al., 2018). This diminishes soil nutrients such as nitrogen, phosphorus, and potassium and disrupts plant nutrient availability (Jiang et al., 2019). Moreover, continuous monoculture stimulates the production of plant autotoxins, particularly phenolic acids, and causes plant diseases (Arafat et al., 2017).

Soil microorganisms play a critical role in the soil ecosystem functions, such as nutrient cycling, organic matter decomposition, toxin breakdown (Khan et al., 2021), and resistance to biotic and abiotic stress (Jin et al., 2015). Soil microbes are valuable indicators of soil fertility and land management practices, as they quickly adapt to environmental and agricultural conditions (Fierer et al., 2021). However, monoculture practices have been found to reduce the diversity and activity of soil microbes (Nayyar et al., 2009), leading to changes in microbial population composition and structure. In intensive sugarcane farming systems that rely on consecutive ratooning, the repeated cultivation of a single crop can lead to the accumulation of crop-specific microbes in the soil (She et al., 2017). This accumulation can stimulate harmful microbes, such as soil-borne pathogens (Wu et al., 2017), while decreasing beneficial microbes, such as *Lysobacter* and *Arthrobacter*. Studies have shown a significant reduction in essential microbial taxa in tea soil subjected to monoculture (Wang et al., 2017). Soil microorganisms are also responsible for producing enzymes that play a crucial role in soil nutrient cycling and organic matter decomposition, among other functions (Bastida et al., 2016). Enzyme activities are closely related to the composition and structure of soil microbial communities (Gracia-Orenes et al., 2013). In sugarcane, ratooning can result in the build-up of crop-specific microbes and the reduction of beneficial soil enzymes. For example, Tayyab et al. (2021) found that ratooning significantly decreased the activities of several enzymes, including urease, invertase, and acid phosphatase. Moreover, they observed that soil microbial diversity and composition were significantly altered, which could decrease soil fertility and nutrient availability. Similarly, Lin et al. (2013) reported that ratooning reduced soil enzyme activities, such as cellulase, polyphenol oxidase, and peroxidase, which are crucial for organic matter decomposition and nutrient cycling. These findings suggest that ratooning practices can negatively impact soil enzyme activities and microbial communities, ultimately affecting soil health and agricultural productivity. However, the exact impact of repeated ratooning on microbial communities and their activities and relationships with soil nutrients and soil enzyme activities in the sugarcane farming system remains poorly understood.

This study aimed to investigate the impact of consecutive ratooning cycles on soil microbial communities and their correlation with changes in soil properties. Previous research suggests that environmental factors play a crucial role in shaping the microbial populations in soil. However, limited research has been conducted to examine the structure and abundance of microbial communities in three consecutive ratooning cycles. To address this gap, we employed various techniques such as high-throughput sequencing, plant morphology analysis, and soil attributes measurement to identify the key ecological factors that influence the composition and distribution of effective microbes in the soil. In addition, we evaluated the 3-year morphological performance of sugarcane to complement our findings. By conducting a comprehensive analysis of soil and plant characteristics, this study aimed to provide insights into the effects of ratooning on land degradation and the potential for sustainable sugarcane cultivation.

## Materials and methods

### Experimental location and plant material

A 3-year field experiment was conducted at the Forage and Breeding station of Guangxi University in Chongzuo, China (22° 38' 06” N, 107° 54' 15” E), a significant sugar-growing site in Guangxi province. Sugarcane genotype ZZ-9 was evaluated in this study using a complete randomized block design. The genotype was sown in a 40-m long row having a 2-m space in between. Plant-to-plant distance was uniformly maintained at 30 cm. The total plot size was 160 m^2^. The same tillage and management techniques were applied to all experimental replicates across the 3-year sugarcane succession, including standardized agronomic practices such as weeding and fertilization. Throughout the ratooning, natural precipitation was the only source of water for the sugarcane.

### Sampling and measurements

In late December, when sugarcane was in the ripening stage, field data were collected for consecutive years (2019–2021). Sugarcane morphological attributes were measured on 30 plants in each replicate, including plant height, stem diameter, internode length, and the number of nodes. Plant height was measured using a meter rod, and the average value was computed after counting the number of nodes per plant. Vernier calipers were used to measure the stem diameter and internode length of every 10th internode from the plant's top, and the mean value was recorded. The rhizosphere soil was collected from the roots, and three subsamples were obtained for further analysis. At harvest, the S-sampling technique was used to collect five soil samples from each plot, which were combined to form a biological replicate. Sterile plastic bags were used to store the soil samples in iceboxes to maintain their freshness, after which they were homogenized and passed through a 2-mm sieve. One part of the samples was air-dried for soil physiochemical analysis, while the second part was stored at a lower temperature (−80°C) for DNA extraction and enzyme activity analysis.

### Soil physicochemical properties and sugarcane yield parameters

The physical–chemical characteristics of soil samples were assessed in accordance with the study by Khan et al. (2022a). The total nitrogen (TN) of the soil was determined using an element analyzer (Thermo Scientific TM, Waltham, MA, USA), and the soil pH was measured using a glass electrode with a soil-to-water ratio of 1:2.5 (Lu, 2000). Using sodium bicarbonate, soil-available phosphorus (AP) was collected and quantified using the molybdenum blue method. An Extech Portable Sucrose Brix Refractometer was used to determine the% Brix (Mid-State Instruments, San Luis Obispo, CA, USA). The theoretical yield of sugarcane was calculated using the formula (Pang et al., 2021):


$$\begin{array}{l} \left(\text{a}\right)\;{\text{Single stalk weight }\left(\text{kg}\right)\;=\;\left(\text{stalk diameter }\left(\text{cm}\right)\right)}^{2}\\ \times \;\left(\mathrm{stalk height}\left(\mathrm{cm}\right)-30\right)\times \;1\;\left({\text{g}/\text{cm}}^{3}\right)\times \;0.7854/1000. \end{array}$$

$$\begin{array}{l} \text{(b) Cane yield }{\text{(t/ha}}^{-\text{1}}\text{) = single stalk weight (kg)}\\ \text{ × stalk numbers (no }{\text{ha}}^{-\text{1}}\text{) 1,000.} \end{array}$$

### Enzymatic activities measurement

Following our previous protocols (Khan et al., 2022a), soil enzymatic activities were estimated accordingly. One portion of the stored soil sample was utilized to determine the soil's enzymatic activity, including acid phosphatase (SACP), catalase (SCAT), and urease (SUE), using soil enzyme kits obtained from Solarbio Science and Technology Co. (Beijing, China), following the manufacturer's instructions.

### DNA extraction, PCR amplification

Using the FASTDNA^TM^ Spin Kit for soil (CO. MP Biomedicals, United States), DNA was extracted from the rhizospheric soil of each sample in accordance with the protocol provided by the manufacturers. Using NanoDrop 2000, the extracted DNA quantity was measured (Thermo Fisher Scientific, Wilmington, United States). Using bacterial primer 799F (5-AACMGGATTAGATACCCKG-3) and reverse primer 1193R (5-ACGTCATCCCCACCTTCC-3), V5–V7 region of 16S rRNA gene was amplified (Khan et al., 2022a). The PCR amplification was conducted using a 25-μL reaction mixture that contained 20 ng of DNA template, 0.5 μL of dNTP, 10 μL of KOD (kodakaraensis) polymerase buffer, 0.25 μL of DNA polymerase, 5 μL of high GC enhancer, and 1.0 μL of each primer (Pang et al., 2019). The first denaturation took place for 5 min at 98°C; then there were 25 rounds of 94°C for 30 s, 52°C for 30 s (annealing), 72°C for 30 s (extension), and 72°C for 10 min (final elongation). The PCR amplification was performed using a Bio-Rad S1000 thermocycler (Bio-Rad Laboratories, CA, United States). The target bands were visualized by 2% agarose gel electrophoresis and extracted using the QIAamp DNA Micro Kit (Qiagen, Valencia, CA, United States). The extracted DNA was used to create DNA libraries using the Illumina TruSeq DNA sample preparation kit (Illumina, San Diego, CA, United States). The Gene Denovo Biotechnology Co., Ltd. (Guangzhou, China) Illumina HiSeq2500 platform was used for high-throughput sequencing of 16S rRNA. The dataset and raw metagenomics generated during this research were deposited in the NCBI Sequence Read Archive (SRA) database with a BioProject ID: PRJNA949169.

### Analysis of sequencing data

To ensure quality, the raw sequences obtained were examined and merged into clean reads using FLASH software. The relevant sample was assigned the clean reads to obtain valid sequences. To analyze the data, the QIIME program (Quantitative Insights into Microbial Ecology v.1.9.0) was utilized. The program received pair-end data as input and used the UCLUST algorithm with a 97% similarity criterion to identify operational taxonomic units (OTUs) from representative sequences (Flynn et al., 2015). The Greengene database was used as a reference database to assign taxonomic labels to each OTU sequence, from phylum to species. The OTU table was used as an input file for further analysis through Microbiome Analyst (Chong et al., 2020), which normalized the data to avoid bias in subsequent analyses. Sequences with a minimum of 4 and a prevalence of 20% were filtered out to remove low variance. A rarefaction curve was constructed using Mothur (v.121.1) to determine the relationship between the number of reads and the number of observed OTUs. For each sample, the relative abundance of distinct taxa was computed, and alpha diversity indices were determined. Additionally, a beta diversity analysis was conducted using the R function to assess the dissimilarities or similarities among the treatments. R (version 3.2.2) was used to conduct a redundancy analysis (RDA) to investigate the impact of soil physiochemical attributes on the abundance of bacterial phyla at the phylum level. DESeq analysis was used to determine the microbial genera that were significantly enriched or depleted with consecutive ratooning (Pang et al., 2022). The correlation between these genera and ecological factors was investigated using Pearson's correlation and Mantel tests. We conducted a non-metric multidimensional scaling (NMDS) analysis using the Bray–Curtis dissimilarity matrices to explore differences and similarities between the three sugarcane fields regarding soil physicochemical properties and enzymatic activities. Additionally, bar graphs were used to visualize the analysis of variance (ANOVA) to estimate significant differences (LSD test, *P* < 0.05) in soil nutrients, enzymes, and plant morphological characters over the 3 years.

## Results

### Morphological and yield parameters

**Figure 1:**
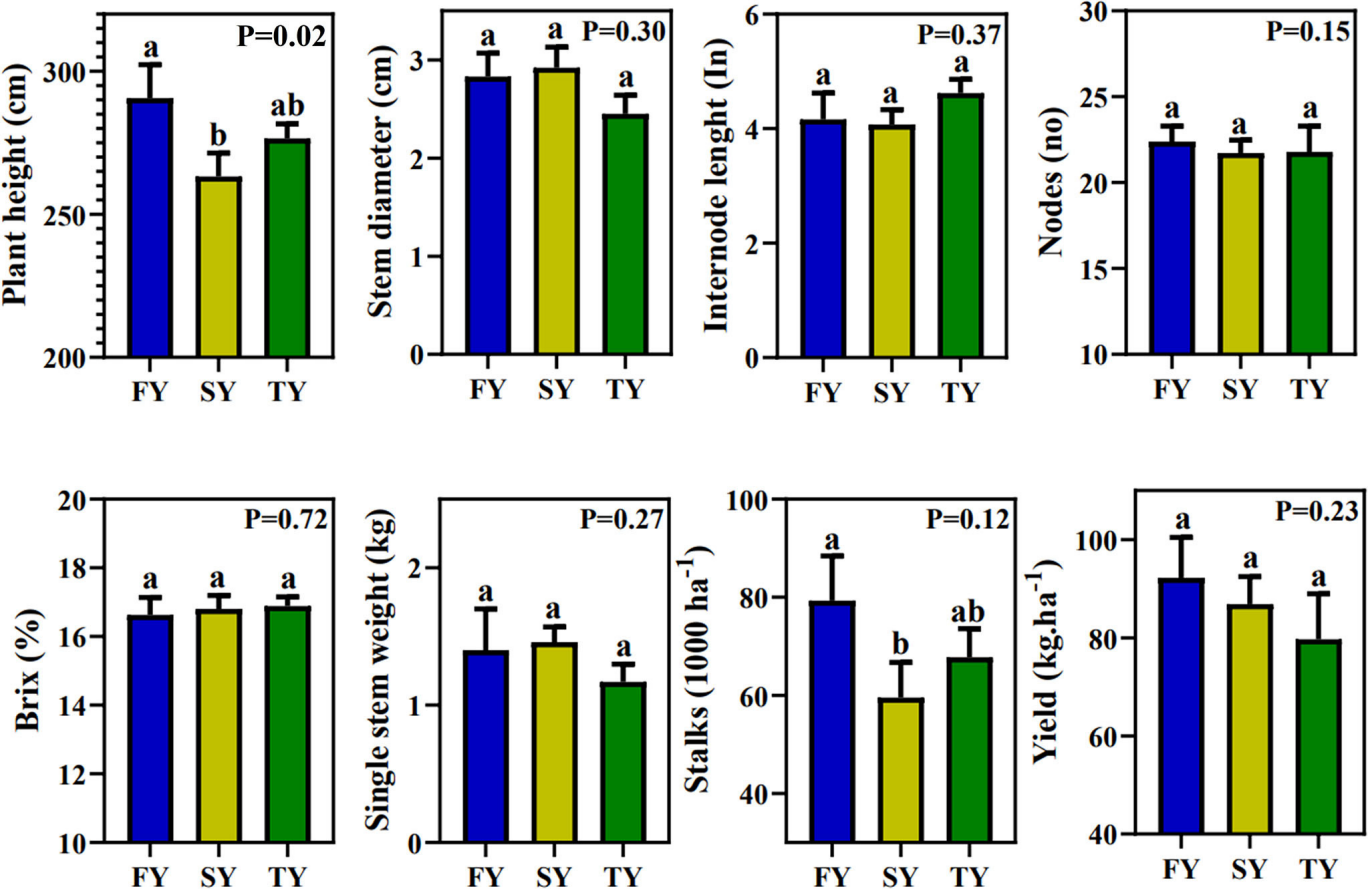
Sugarcane field performance in consecutive ratooning. Different letters indicate significant differences among the ratooning sugarcane (LSD test, *P* < 0.05). FY, first year; SY, second year; TY, third year.

Figure 1 displays the field agronomic performance of sugarcane during consecutive ratooning. However, except for plant height, all the other parameters were statistically nonsignificant. Plant height decreased in SY and TY by 9.23% and 4.65%, respectively. For the yield parameter, SSW first increased by 4.28% in SY and then decreased by 16% in TY. The number of stalks per hectare decreased by 24.9 and 14.4% in SY and TY, respectively. The theoretical yield per hectare decreased by 22.61 and 29.01% in SY and TY, respectively (Figure 1). Generally, all the morphological traits exhibited a decrease in performance with ratooning year.

### Soil parameters and enzymatic activities

**Figure 2:**
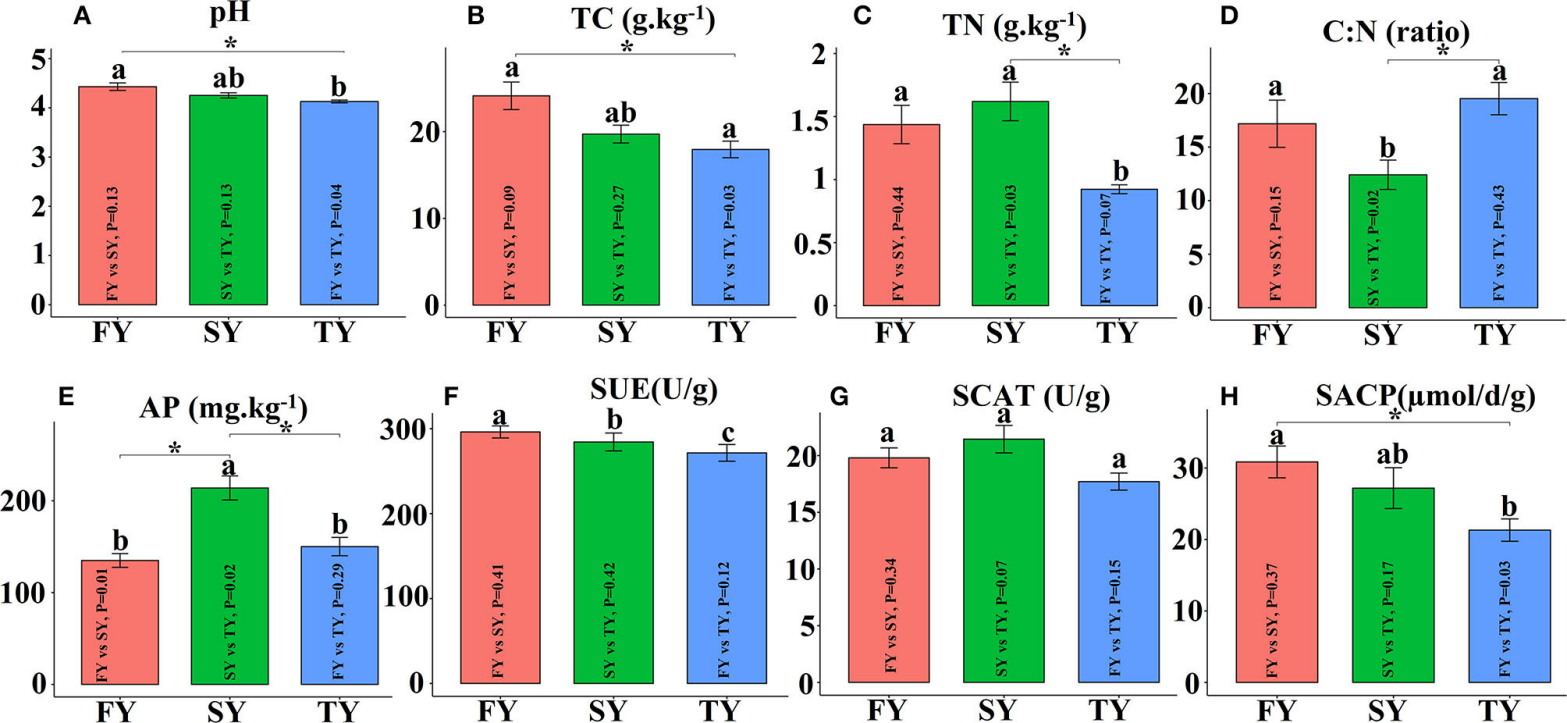
Soil physiochemical properties and enzymatic activities in consecutive ratooning. Different letters indicate significant differences among the ratooning sugarcane (LSD test, *P* < 0.05). FY, first year; SY, second year; TY, third year. **(A)** pH, potential of hydrogen; **(B)** TC, total carbon; **(C)** TN, total nitrogen; **(D)** C:N, ratio of carbon and nitrogen; **(E)** AP, available phosphorous; **(F)** SUE, Soil urease; **(G)** SCAT, soil catalase; **(H)** SACP, soil acid phosphatase.

**Figure 3:**
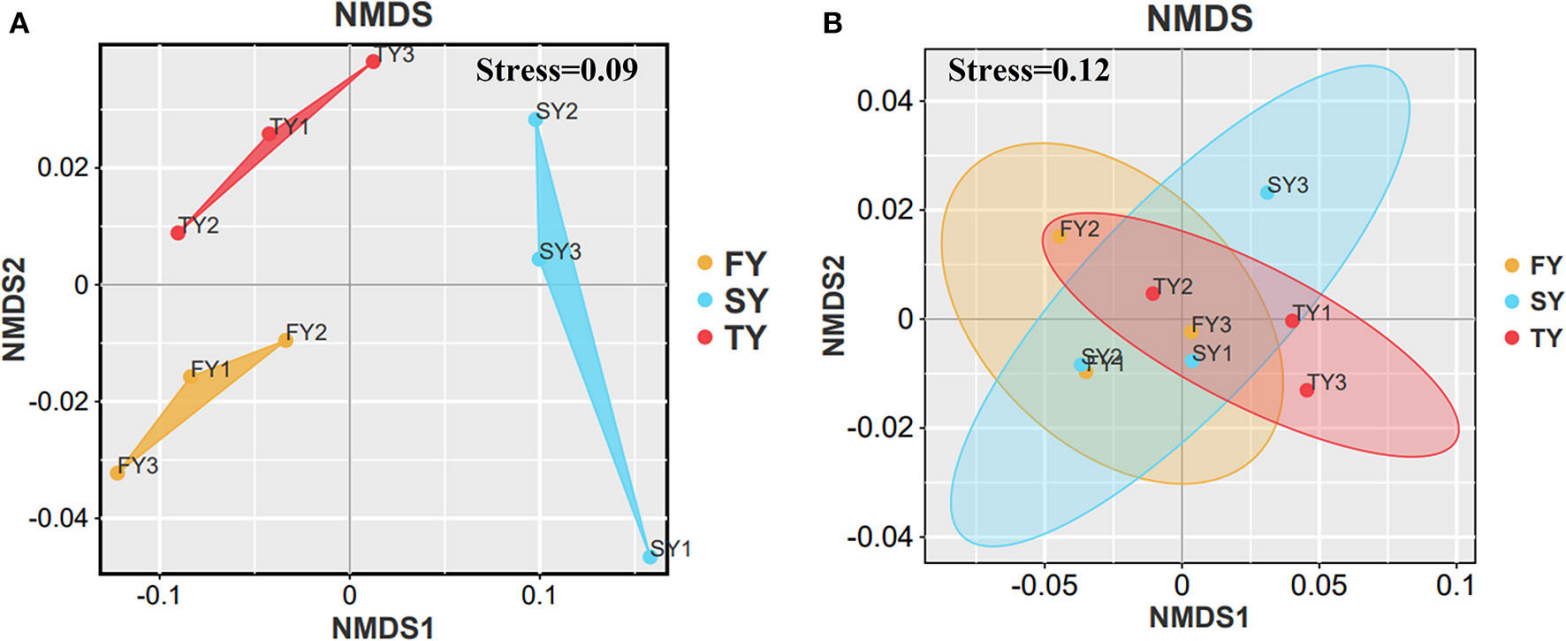
NMDS analysis of soil nutrients and soil enzymes in three consecutive crop cycles calculated on Bray–Curtis. **(A)** NMDS analysis of soil nutrients in consecutive ratooning and **(B)** NMDS analysis of soil enzymes in consecutive ratooning.

Figure 2 displays the soil physiochemical properties during the ratooning sugarcane. Consecutive ratooning resulted in soil acidity and a significant decrease in vital soil nutrients, including TC and TN (Figures 2B, C). Soil enzymatic activities also decreased during the third ratooning; however, their effect was statistically non-significant. Soil AP and C:N ratios exhibited an imbalance in their composition during the ratooning years (Figures 2D, E). Furthermore, non-metric multidimensional scaling (NMDS) confirmed the matrices of dissimilarity (ANOSIM, *R* = 0.539) and statistically significant (*P* = 0.005) between the ratooning years and soil nutrients (Figure 3A). Soil enzymatic activities also showed a trend of decrease during the consecutive ratooning; however, the matrices of dissimilarity (NMDS) were statistically not significant (*R* = 0.15, *P* = 0.482) (Figure 3B). The soil ACP decreased significantly in the third ratooning year compared to the first ratooning. Although the other two enzymes, SUE and SCAT, did not exhibit significant differences, it can be speculated that consecutive ratooning causes a decrease in soil enzymes involved in C, N, and P cycling. Furthermore, a strong positive and significant correlation was observed between soil enzymes and nutrients. Soil pH was positively and significantly correlated with SACP and SUE (Supplementary Figure 1A).

### Sequencing details and microbial diversity analysis

**Figure 4:**
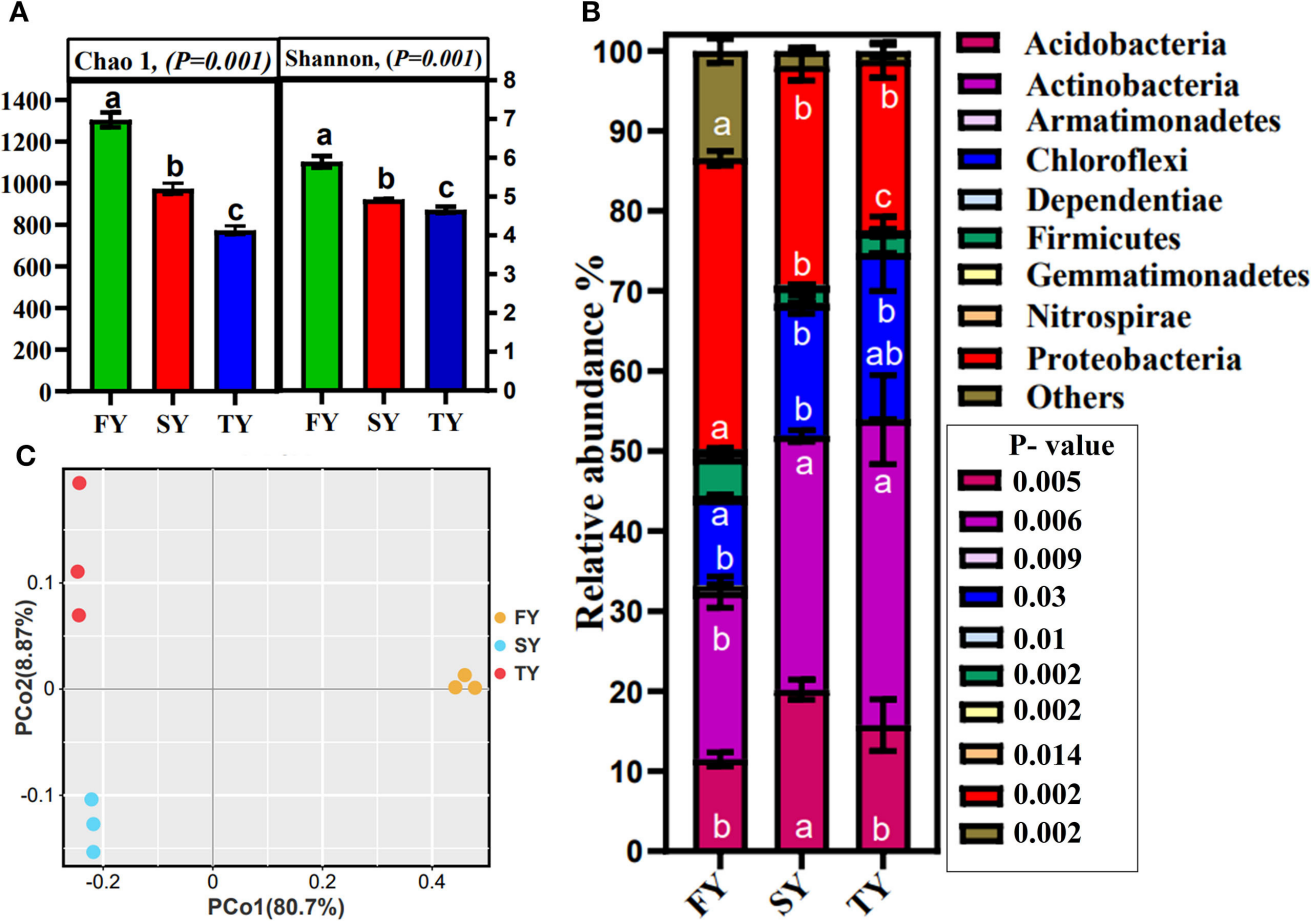
Bacterial alpha diversity indices Shannon and Chao 1 **(A)**. Relative abundance of major bacterial phyla of sugarcane rhizosphere in consecutive ratooning **(B)**. Different letters indicate significant differences among the ratooning sugarcane (LSD test, *P* < 0.05). Bacterial beta diversity analysis and principal coordinate analysis (PCoA) of 3-year samples based on Bray–Curtis distances of replicated samples during consecutive ratooning **(C)**.

A total of 420,447 bacterial reads with an average of 46,717 were obtained from the soil samples. Bacterial OTUs detected in the 3-year ratooning crop varied among the samples, including 325, 235, and 249 OTUs in FY, SY, and TY, respectively (Supplementary Figure 1B). The rarefaction curve displays enough depth for sequencing (Supplementary Figure 1C). Bacterial diversity (Shannon) and richness (Chao1) decreased significantly during the consecutive ratooning (Figure 4A). The results indicate that soil microbial diversity was severely affected during consecutive ratooning. The rhizosphere bacterial community was overall dominated by Actinobacteria (30%), Proteobacteria (28%), Chloroflexi (16%), and Acidobacteria (15%), among the others. The relative abundances of major phylum and their major classes are given in Supplementary Figure 2. Interestingly, the consecutive ratooning significantly affected the first three important bacterial phyla by decreasing or increasing their relative abundance. An increase of 81.8% in SY and 15% in TY was observed in the relative abundance of Acidobacteria compared to FY (Figure 4B). Similarly, a 55% increase in SY and 90% in TY was observed for Actinobacteria compared to FY. An increase of 60% in SY and 100% in TY was observed for Chloroflexi compared to FY. Firmicutes decreased relative abundances by 58% in SY and 45% in TY compared to FY. The essential bacterial phyla, Proteobacteria, decreased by 25% and 41% in SY and TY, respectively. A similar trend was observed in the relative abundance of important bacterial genera, including *Sphingomonas, Burkholderia, Bradyrhizobium*, and *Bacillus* among others, and showed alteration in their relative abundances. For instance, the relative abundance of *Sphingomonas* decreased by 87% and 84% in SY and TY, respectively (Supplementary Figure 3A).

Principal coordinate analysis was carried out using Bray–Curtis distances to estimate beta diversity. The results displayed that bacterial communities in consecutive ratooning have different patterns (ANOSIM, *R* = 0.84, *P* = 0.009). Furthermore, the first two axes explained 89.57% of the total variation in bacterial communities, with PCoA1 explaining 80.7% variation and PCoA2 explaining 8.87% variation (Figure 4C).

### Effect of consecutive ratooning on bacterial community composition

**Figure 5:**
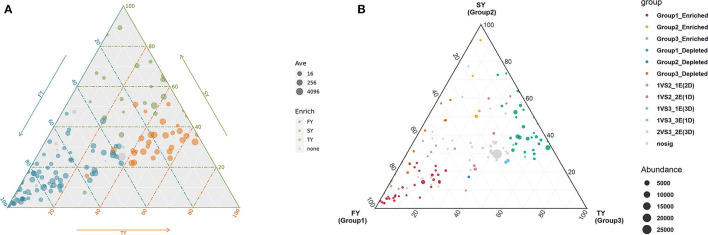
Ternary plot analysis showing the enriched and depleted genera for bacterial community composition during consecutive ratooning **(A, B)**. The position of the point corresponds to its relative abundance relating to each group. Each point corresponds to enriched or depleted genera. FY, first year; SY, second year; TY, third year. For reference of each enriched or depleted genera readers are referred to Supplementary Table 1 of this article.

**Figure 6:**
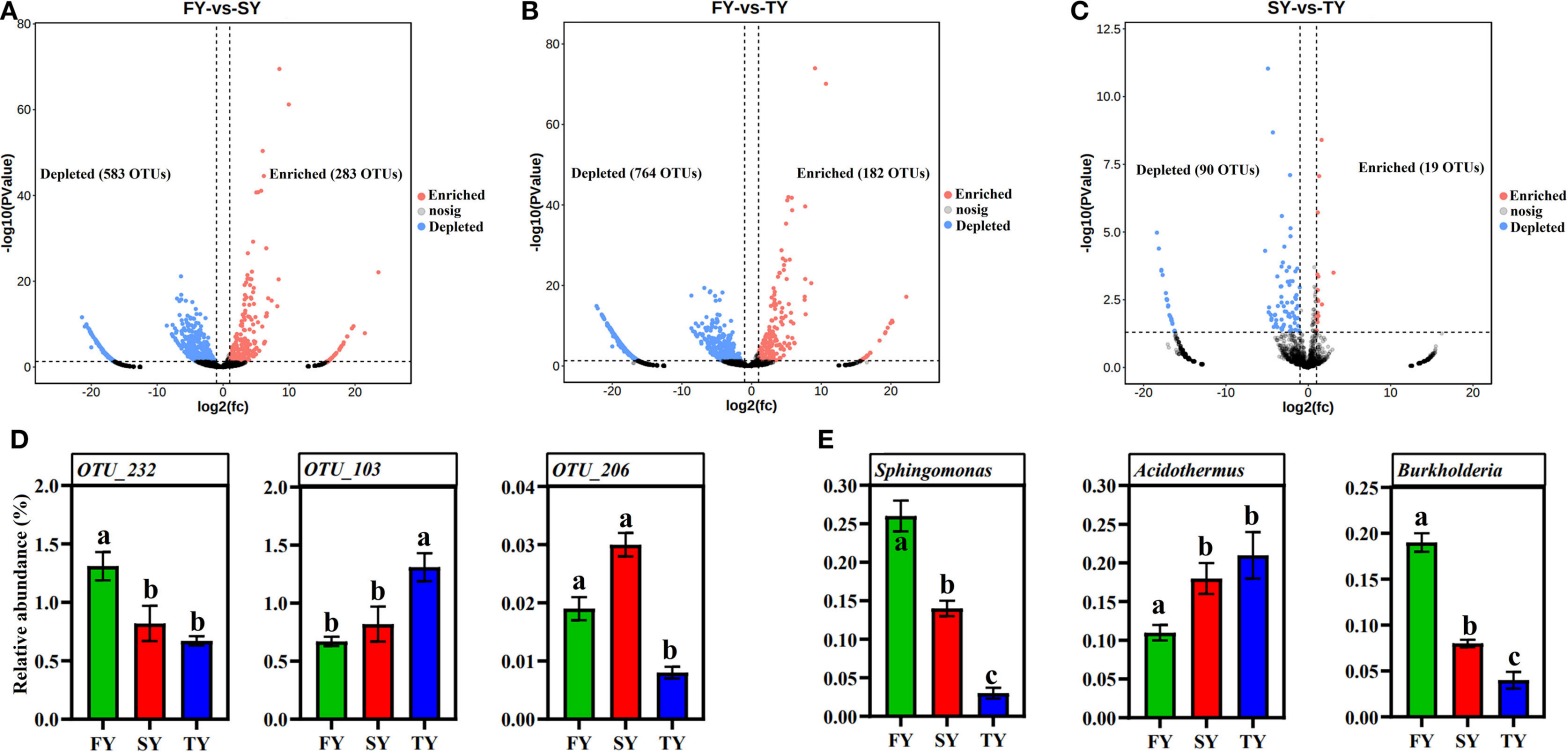
Volcano plots showing differentially abundant OTUs during consecutive ratooning sugarcane (FDR corrected *P* < 0.01). OTUs enriched are colored in red, OTUs depleted are colored in blue, and gray represents non-differentially abundant OTUs **(A–C)**. The relative abundances of OTU_232, OTU_103, and OTU_206 **(D)**. The relative abundance of *Sphingomonas, Acidothermus*, and *Burkholderia* in each ratooning year **(E)**. FY, first year; SY, second year; TY, third year.

A ternary plot analysis was carried out to unveil the dynamic effects of consecutive ratooning on soil bacterial populations over consecutive years. In general, the combined analysis of all samples revealed that with consecutive ratooning the majority of bacterial genera, including *Acidibacter, Acidothermus, Sphingomonas, Burkholderia, Bradyrhizobium*, and *Bryobacter* were abundant and enriched. The specific bacterial genera were identified, either enriched or depleted because of ratooning (Figure 5). The detail about each genus and its enrichment or depletion is given in Supplementary Table 1. In-depth differential abundance analysis with the GLM approach found enriched and depleted OTUs in the rhizosphere samples. Compared to FY, SY, and TY exhibited 283 and 182 enriched OTUs, while depleting 583 and 764 OTUs, respectively (Figures 6A, B). Compared to SY, 19 OTUs were enriched and 90 were depleted in TY (Figure 6C). In specific, OTU_1232, OTU_103, and OTU_206 were identified as the most abundant OTUs among all the samples (Supplementary Figure 3B), recognized as *Sphingomonas, Acidothermus*, and *Burkholderia*, respectively (Figure 6D). The relative abundance of OTU_232 (*P* = 0.004) and OTU_103 (*P* = 0.007) was statistically significant, while that of OTU_206 was non-significant (*P* = 0.15). The interesting phenomenon observed here was, unlike the other two OTUs the relative abundance of OTU_103 increased with ratooning sugarcane. The relative abundance of *Sphingomonas* (*P* = 0.004) and *Burkholderia* (*P* = 0.15) was higher in FY and later on decreased with ratooning (Figure 6E), while the relative abundance of *Acidothermus* increased with ratooning (*P* = 0.007).

### Microbial community structure and soil variables

**Figure 7:**
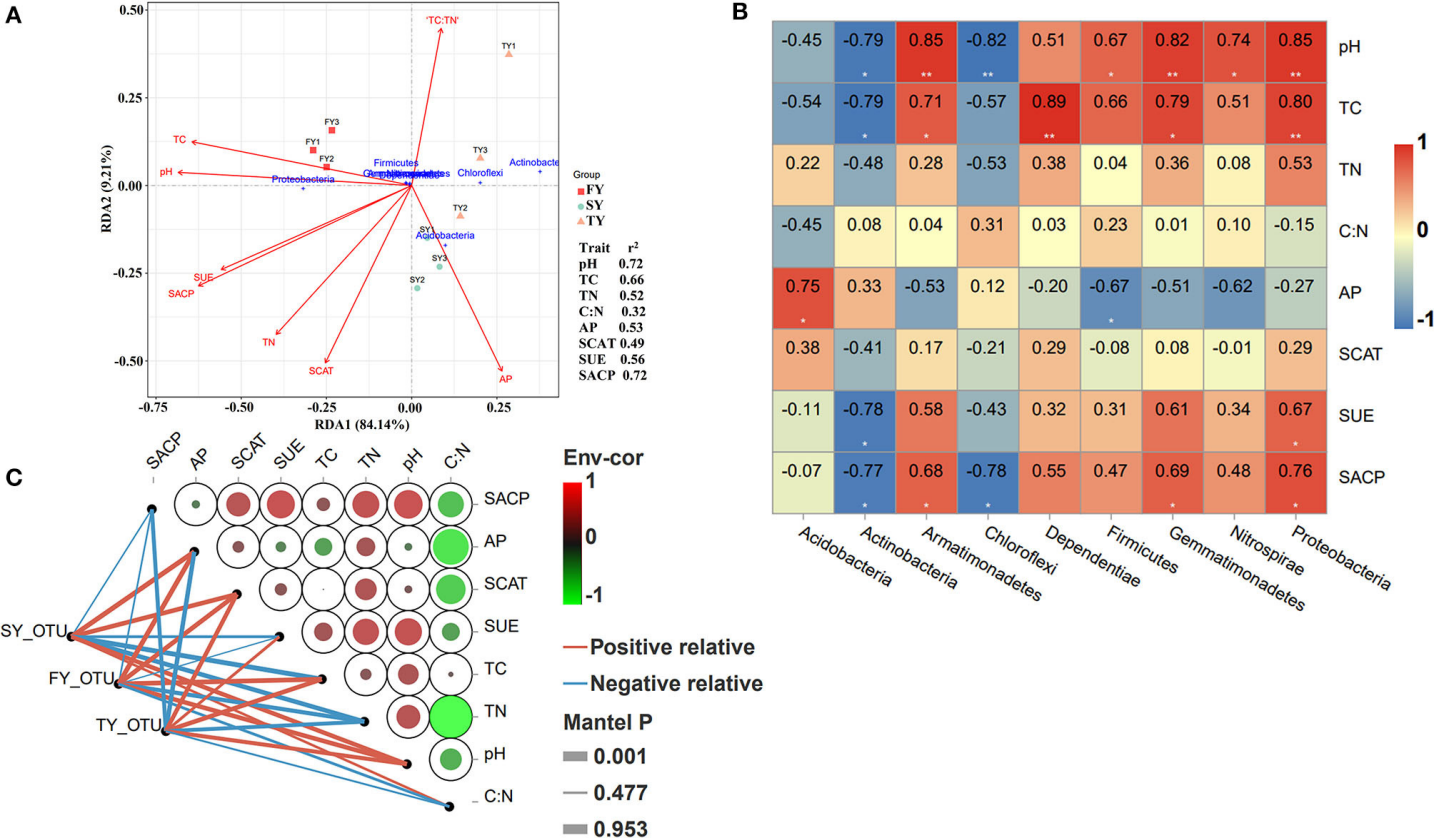
RDA analysis of soil nutrients, enzymes, and major bacterial phyla. Right side values indicate the *r*^2^ of each environmental factor. FY, first year; SY, second year; TY, third year **(A)**. Pearson's correlation analysis between major bacterial phyla and soil physiochemical properties. **shows the significance level at *P* < 0.01 and *shows the significance level at a *P-*value of < 0.05. Red represents a strong positive correlation, pale yellow represents a weak correlation, and blue color represents a strong negative correlation **(B)**. Pairwise comparisons of ecological determinants are demonstrated with a color gradient showing Pearson's correlation coefficients. The abundance of microbial phyla determined using DESeq analysis (based on the generalized linear binomial negative model) in response to monoculture was correlated with each ecological factor using Mantel tests. Edge width corresponds to the Mantel's r statistic for the corresponding distance correlations, and edge color denotes the statistical significance **(C)**. FY, first year; SY, second year; TY, third year.

Redundancy analysis (RDA) was performed to explore the relationship of soil variables with the bacterial population in consecutive ratooning (Figure 7A). The analysis confirmed that TC, pH, C:N, SUE, SACP, SCAT, and AP explained 93% of the overall variation in ratooning bacterial communities. RDA1 explains 84% variation, while RDA2 explains 9% variation. Phylum Proteobacteria was an important factor associated with soil variables during the first year. The important factors affecting the bacterial population were investigated through the Mantel test, and it found that soil pH (*R* = 0.72, *P* = 0.016), TC (*R* = 0.66, *P* = 0.03), and SACP (*R* = 0.72, *P* = 0.025) were the main factors to influence soil microbial communities (Figure 7A). Furthermore, correlation analysis (Pearson's) revealed that phylum Proteobacteria, Nitrospirae, Gemmatimonadetes, and Firmicutes were positively and significantly correlated with soil pH and TC (Figure 7B). Phylum Actinobacteria, Chloroflexi, and Acidobacteria were negatively correlated with soil pH and TC.

The Mantel test along with Pearson's correlation analysis was used to unveil the influence of soil microbial communities and environmental factors during ratooning sugarcane (Figure 7C). The results revealed that SCAT, SUE, TN, and pH were the main drivers of depleting soil bacterial populations. Bacterial abundance in FY was positively and significantly correlated with AP, SCAT, TC, and pH. However, an imbalance in correlation was observed in bacterial abundance during the SY and TY with soil variables.

## Discussion

Sugarcane ratooning is widespread in many sugarcane-producing regions as it allows multiple harvests from a single planting; however, ratooning usually leads to degradation by negatively affecting soil fertility. This ultimately causes soil acidification and increases the likelihood of soil-borne diseases (Liu et al., 2019), which adversely affect the yield of sugarcane. This issue is widespread in sugarcane-growing areas, notably in Australia, New Guinea, Fiji, Papua, and China (Lin et al., 2013). Despite extensive research into the effects of soil management on soil acidity and fertility, the impact of consecutive sugarcane ratooning on microbial community composition and changes along with the soil parameters still needs to be fully understood. The current study highlights the impact of consecutive ratooning on soil microbial structure, pH, and soil nutrients and enzymes. Additionally, the study explores how soil properties vary in association with essential microbial taxa.

### Sugarcane ratooning, soil physiochemical properties, and enzymatic activities

Soil nutrients play a crucial role in soil ecology by conserving soil water and providing essential micronutrients, as well as nitrogen, phosphorus, and sulfur, for microorganisms and plants (Obalum et al., 2017), thus helping to maintain soil fertility and promote crop production. The current study found that ratooning reduced soil pH and changed the soil nutritional pattern, such as available potassium, total carbon, available phosphorus, and total nitrogen. Ratooning-induced soil acidity is likely due to the accumulation of allelochemicals (Ehrenfeld et al., 2005) or sugarcane residues (Sampietro and Vattuone, 2006). It has been suggested that reduced organic matter input and constant use of chemical fertilizers rather than organic fertilizers could be responsible for the decline in soil nutrients (Sampietro et al., 2007). The use of ammonium-N fertilizers as acid inputs and alkali remediation from the plant's absorption could cause an acidic environment in a sugarcane cropping system. The Guangxi region experiences a heavy input of urea fertilizers to attain maximum yield (Khan et al., 2022a) which might be a possible reason for soil acidity in the experimental area. The present study indicated the negative impact of ratooning on soil nutrients. The findings of this research are well supported by Tayyab et al. (2021), who investigated the primary effect of sugarcane monoculture on soil nutrients and enzymes in three different fields.

Soil enzymes play a crucial role in maintaining soil fertility by catalyzing important biochemical reactions, such as nutrient cycling and organic and xenobiotic nutrient degradation, making them sensitive indicators of soil fertility (Khan et al., 2022b). The decrease in enzymes exhibited that C and N cycles in SY and TY compared to FY suggests that ratooning is the most significant factor contributing to the decline in soil N, C turnover, and fertility. Lower enzyme activities observed in SY and TY indicate a reduced turnover rate of soil N and C, leading to decreased soil fertility. These findings agree with previous research, which suggests that the continuous cultivation of various crops decreases soil enzyme activities (Chen et al., 2018) and negatively impacts nutrient turnover and soil fertility (Song et al., 2018). In this study, during the second ratooning (SY), the soil nutrients were observed to be decreasing despite increased SCAT enzyme activity. SCAT is involved in the cycling of nitrogen, carbon, and sulfur. Possible explanations for increased enzyme activity and low nutrients include temperature and the presence of other soil enzymes (Wang et al., 2017), as it is a complex phenomenon and is controlled by many factors. While it is possible to have low nutrient concentrations in the soil despite high enzyme activity, more research is needed to fully understand the underlying mechanisms.

### Soil pH is essential for soil enzymes and N, C, and P cycles

Soil enzymes are essential in maintaining soil fertility, and their activities are influenced by soil pH. In the present study, reduced soil enzyme activity in ratooning results in soil acidity. Soil pH has a direct impact on soil enzyme activity by altering the biochemical reaction rates of enzymes (Dick et al., 2000). In the present study, the decrease in soil enzyme activity due to ratooning is due to soil acidification. Evidence exists that soil pH influences soil enzymes and bacterial communities in paired sugarcane fields with short- or long-term cultivation histories (Bigott et al., 2019). Our study, however, highlights the effect of three consecutive ratooning as a primary factor influencing soil pH.

### Soil pH is an important factor influencing soil microbial community structure

Soil pH and nutrient acquisition (TC and AP) are widely used indicators to measure soil quality, as these factors possess a quick response to soil variations (Khan et al., 2021). The practice of ratooning creates an acidic environment characterized by low pH and fertility. Acidity in ratooning sugarcane is due to the release of organic acids from decomposing sugarcane residues and the depletion of soil calcium and magnesium (de Souza et al., 2023). This environment can lead to a decrease in soil bacterial diversity and nutrient availability, as many bacterial groups are sensitive to changes in soil pH. In the current research, we confined such results and are well supported by Tayyab et al. (2021). Shifts in microbial diversity are valuable indicators of soil function degradation or restoration (Wagg et al., 2019), and low soil pH can have a significant effect on bacterial diversity in sugarcane fields (Tayyab et al., 2021). In this study, ratooning decreased bacterial diversity, supporting the previous research on tea monoculture (Arafat et al., 2017). A change in soil microbial diversity is associated with decreased soil pH in a monoculture system (Singh et al., 2014). Our present findings of ratooning sugarcane and decreased soil pH are supported by Tayyab et al. (2021), who investigated decreased bacterial diversity in sugarcane fields with different soil pH levels. Ratooning-induced soil acidification is an important factor in reducing the abundance of Proteobacteria and potentially affecting plant growth. This supports our research, where the abundance of Proteobacteria and soil pH showed a positive correlation (Supplementary Figure 1A). Ratooning, which is a type of continuous monoculture, has been found to influence soil microbial communities in monoculture systems such as peanut (Chen et al., 2012), vanilla (Xiong et al., 2015), notoginseng (Tan et al., 2017), and tea (Arafat et al., 2017), and these shifts may be speculated to root exudates, soil chemical composition, and plant residues resulting from sugarcane cropping. Liming may be necessary to overcome low soil pH in sugarcane fields (Pang et al., 2019) to increase the soil pH and reduce the potential for nutrient deficiencies and toxicity. For this purpose, soil testing is recommended to help determine the appropriate liming rate based on soil pH, soil type, and other factors.

### Sugarcane ratooning depletes/enriches essential bacterial genera

Through pyrosequencing of ecological data, we identified several important microbial genera that resulted in depletion or enrichment because of ratooning. While we can only speculate on the ecological role of these taxa based on previous research (Hartmann et al., 2015) in cropping systems, understanding how ratooning affects harmful, beneficial, and disease-causing microbial taxa is invaluable. For instance, with increasing ratooning time, several bacterial genera, including *Nitrospira, Pseudomonas, Streptomyces, Actinomadura*, and *Sphingomona*s, which control many plant diseases (Patel et al., 2019), control nitrogen cycle (Green et al., 2010), reduce heavy metal pollution, enhance plant resistance to abiotic stress, and have high phosphorus content (Akar et al., 2006), significantly decreased. In the present study, ratooning resulted in the depletion of many important bacterial genera related to plant growth promotion, nitrogen cycle, suppressing plant diseases, and recycling carbon, and nitrogen. The top three abundant OTUs in this study belonged to *Sphingomonas, Acidothermus*, and *Burkholderia* (Figure 6). Ratooning sugarcane significantly (*P* < 0.05) reduced the abundance of *Sphingomonas* and *Burkholderia*. Both of these bacteria are involved in nitrogen fixation in soil, and alteration in their abundance can cause noticeable changes in plant growth and soil nutrient composition (Pang et al., 2019).

Interestingly, we observed an increased relative abundance of *Acidothermus* bacteria with increased ratooning (Figure 6E). *Acidothermus* bacteria are known to be acid tolerant and can thrive best in soil with a lower pH (Ogola et al., 2021). However, in an acidic environment, many nutrients are less available to plants, ultimately leading to nutrient depletion. This suggests that ratooning in sugarcane is an important factor that induces acidity, which alters the composition of bacterial communities and ultimately affects soil nutrient composition. An alternative for boosting the abundance of these important microbes might be using environmentally friendly farming methods such as biologically produced fertilizers (Wang et al., 2015). By manipulating soil nutrient levels in the ratooning crop through liming or the use of biofertilizers, it is possible to enhance the structure and activity of these microbial communities, which could lead to the establishment of sustainable sugarcane planting systems. Currently, we are in the process of identifying the effect of biofertilizers on bacterial community composition in ratooning sugarcane.

## Conclusion

Ratooning in sugarcane resulted in an acidic environment, less soil nutrients such as TN, TC, and AP, and several important enzymes involved in nutrient cycling. Ratooning not only increased soil acidity but also resulted in significant changes in the bacterial community. The relative abundance of Acidobacteria, Actinobacteria, and Chloroflexi increased significantly during the ratooning of sugarcane. The important bacterial phylum (Proteobacteria) which contains many plants growth-promoting rhizobacteria decreased significantly and hence affected sugarcane yield. Ratooning influenced soil microbial structure and related properties due to alteration in soil physiochemical properties. Different bacterial taxa respond differently to ratooning, of which the plant-beneficial taxa *Sphingomonas* and *Burkholderia* were reduced significantly with increasing crop time. Therefore, using certain agricultural practices such as liming or biofertilizers can help reduce soil acidity and improve soil fertility, ultimately slowing down land degradation and enhancing the structure and activities of the soil microbial community. This will serve as a useful step in forwarding toward a sustainable sugarcane farming system especially ratooning sugarcane.

## Data availability statement

The datasets presented in this study can be found in online repositories. The names of the repository/repositories and accession number(s) can be found below: https://www.ncbi.nlm.nih.gov/, PRJNA949169.

## Author contributions

MZ and AK conceived the main idea of this manuscript. AK wrote the manuscript. MZ, IA, and MA revised the manuscript and provided suggestions. AK, YW, MA, and IA analyzed the data. MZ supervised the manuscript and provided financial support. All the authors contributed intellectually to this manuscript and assisted in manuscript preparation.

## Supplementary material

Supplementary Figure 1 Heatmap correlation analysis of soil enzymes with soil nutrients. ^*^Shows the significance level at a *P*-value of < 0.05. Red color represents a strong positive correlation, white color represents a weak correlation and blue color represents a strong negative correlation **(A)**. Venn diagram analysis illustrating the number of unique and shared OTUs in each year **(B)**. Rarefaction curve depicting species richness and sequences per sample **(C)**. FY, first year; SY, second year; TY, third year.

Supplementary Figure 2 Overall relative abundance of bacterial phyla in rhizosphere soil of consecutive ratooning sugarcane, with a major abundance of classes Proteobacteria, Acidobacteria, and Chloroflexi, the top three phyla.

Supplementary Figure 3 Relative abundance of major bacterial phyla and genera of sugarcane rhizosphere in consecutive ratooning. **(A)** Different letters indicate significant differences among the ratooning sugarcane (LSD test, *P* < 0.05). **(B)** Relative abundance of top 20 OTUs in the rhizosphere samples of ratooning sugarcane. FY, first year; SY, second year; TY, third year.
