# Supplementary material for: Dynamics of rhizosphere bacterial communities and soil physiochemical properties in response to consecutive ratooning of sugarcane
Source: Front Microbiol. 2023 Jul 10;14:1197246. doi: 10.3389/fmicb.2023.1197246 (PMC10364612; doi:10.3389/fmicb.2023.1197246)
Supplement: Supplementary file 4 [file Table_1.DOCX]

Table S1. List of enriched and depleted genera in sugarcane rhizosphere bacterial community during the consecutive ratooning.

| Genus | FY | SY | TY | group |
| --- | --- | --- | --- | --- |
| FCPS473 | 541.2 | 275.5 | 473.1 | ABAE |
| Acidisphaera | 350.6 | 195.8 | 297.9 | ABAE |
| Piscinibacter | 8.3 | 1.6 | 6.1 | ABAE |
| Gaiella | 37.7 | 18.9 | 32.4 | ABAE |
| Devosia | 4.0 | 14.5 | 5.8 | ABBE |
| Actinomycetospora | 0.5 | 6.0 | 3.3 | ABBE |
| Terrabacter | 1.9 | 11.8 | 6.9 | ABBE |
| Acidipila | 76.4 | 133.0 | 115.9 | ABBE |
| JG30a_KF_32 | 5.8 | 0.8 | 0.0 | ACAE |
| Desulfosporosinus | 5.5 | 0.7 | 0.4 | ACAE |
| Acidocella | 6.8 | 1.6 | 0.0 | ACAE |
| Allorhizobium | 42.5 | 6.0 | 0.7 | ACAE |
| Sphingobacterium | 4.6 | 1.2 | 0.0 | ACAE |
| GAS113 | 11.2 | 5.2 | 2.0 | ACAE |
| Pseudarthrobacter | 6.0 | 1.9 | 0.0 | ACAE |
| Prauserella | 8.1 | 3.2 | 0.0 | ACAE |
| Chryseobacterium | 19.1 | 12.0 | 4.1 | ACAE |
| Alcaligenes | 4.5 | 1.9 | 0.0 | ACAE |
| Phenylobacterium | 87.2 | 61.6 | 40.7 | ACAE |
| Anaeromyxobacter | 12.2 | 6.4 | 2.3 | ACAE |
| Methylobacterium | 6.3 | 3.9 | 0.5 | ACAE |
| Ralstonia | 9.5 | 19.1 | 26.5 | ACCE |
| Weissella | 14.3 | 26.5 | 53.6 | ACCE |
| Acetobacter | 2.4 | 10.8 | 0.8 | BCBE |
| Acidothermus | 244.0 | 4496.9 | 8858 | Group1_Depleted |
| Dyella | 43.2 | 743.7 | 540.6 | Group1_Depleted |
| Terracidiphilus | 8.5 | 107.5 | 32.0 | Group1_Depleted |
| Occallatibacter | 531.8 | 3697.4 | 2835 | Group1_Depleted |
| Bryobacter | 348.3 | 1474.8 | 2068. | Group1_Depleted |
| Jatrophihabitans | 54.4 | 496.6 | 723.8 | Group1_Depleted |
| Chujaibacter | 38.6 | 472.7 | 757.2 | Group1_Depleted |
| Bradyrhizobium | 209.8 | 1215.4 | 1308.7 | Group1_Depleted |
| Edaphobacter | 0.7 | 58.0 | 32.0 | Group1_Depleted |
| Nocardia | 0.0 | 46.9 | 81.0 | Group1_Depleted |
| Acidicaldus | 30.5 | 214.6 | 366.5 | Group1_Depleted |
| Pseudolabrys | 0.0 | 144.6 | 82.0 | Group1_Depleted |
| Conexibacter | 946.0 | 2833.7 | 3453.8 | Group1_Depleted |
| Granulicella | 47.1 | 213.8 | 122.8 | Group1_Depleted |
| Acidibacter | 593.2 | 1846.9 | 2343.8 | Group1_Depleted |
| Geodermatophilus | 34.9 | 119.1 | 220.9 | Group1_Depleted |
| Serratia | 0.0 | 19.4 | 11.2 | Group1_Depleted |
| Solibacillus | 1.5 | 25.6 | 20.6 | Group1_Depleted |
| Roseiarcus | 79.5 | 236.8 | 483.2 | Group1_Depleted |
| Marmoricola | 0.2 | 13.8 | 4.7 | Group1_Depleted |
| Actinocatenispora | 116.5 | 264.6 | 309.9 | Group1_Depleted |
| Intrasporangium | 3.2 | 18.8 | 15.8 | Group1_Depleted |
| Leifsonia | 3.3 | 18.9 | 26.7 | Group1_Depleted |
| Vulgatibacter | 0.0 | 6.0 | 6.9 | Group1_Depleted |
| Streptacidiphilus | 45.3 | 108.8 | 248.9 | Group1_Depleted |
| Reyranella | 49.3 | 109.8 | 152.9 | Group1_Depleted |
| Gemmatirosa | 0.0 | 5.0 | 5.1 | Group1_Depleted |
| Tumebacillus | 41.5 | 91.0 | 164.5 | Group1_Depleted |
| Kribbella | 0.5 | 5.0 | 6.7 | Group1_Depleted |
| Mycobacterium | 555.0 | 925.3 | 1439.0 | Group1_Depleted |
| Candidatus_Ovatusbacter | 162.9 | 6.8 | 0.8 | Group1_Enriched |
| Sphingomonas | 3988.0 | 814.2 | 881.2 | Group1_Enriched |
| Cohnella | 136.5 | 6.5 | 10.7 | Group1_Enriched |
| Pullulanibacillus | 102.6 | 7.6 | 7.3 | Group1_Enriched |
| Inquilinus | 216.4 | 30.5 | 19.4 | Group1_Enriched |
| Pseudonocardia | 658.5 | 187.5 | 183.3 | Group1_Enriched |
| Hyphomicrobium | 92.8 | 2.8 | 2.9 | Group1_Enriched |
| Paenibacillus | 204.9 | 49.6 | 63.0 | Group1_Enriched |
| Mesorhizobium | 57.5 | 3.4 | 1.6 | Group1_Enriched |
| Rubrobacter | 42.8 | 1.5 | 0.8 | Group1_Enriched |
| Candidatus_Nitrosoglobus | 42.2 | 2.5 | 1.9 | Group1_Enriched |
| Alicyclobacillus | 214.2 | 48.5 | 79.8 | Group1_Enriched |
| Streptomyces | 358.3 | 84.7 | 132.5 | Group1_Enriched |
| 1921_3 | 257.5 | 94.2 | 105.2 | Group1_Enriched |
| Kroppenstedtia | 22.1 | 1.7 | 5.3 | Group1_Enriched |
| Vibrio | 12.4 | 0.4 | 0.4 | Group1_Enriched |
| Pelomonas | 16.6 | 1.3 | 1.5 | Group1_Enriched |
| Romboutsia | 16.7 | 1.1 | 1.1 | Group1_Enriched |
| Clostridium_sensu_stricto_12 | 27.0 | 4.6 | 4.4 | Group1_Enriched |
| Sporichthya | 77.8 | 23.3 | 26.5 | Group1_Enriched |
| Candidatus_Jidaibacter | 13.7 | 0.8 | 0.5 | Group1_Enriched |
| Staphylococcus | 27.0 | 4.7 | 8.3 | Group1_Enriched |
| Coxiella | 11.3 | 0.8 | 0.4 | Group1_Enriched |
| Phaselicystis | 39.9 | 14.5 | 4.3 | Group1_Enriched |
| Dokdonella | 25.9 | 7.7 | 4.0 | Group1_Enriched |
| Haloplasma | 10.5 | 0.9 | 0.7 | Group1_Enriched |
| Thermoactinomyces | 9.8 | 0.8 | 1.4 | Group1_Enriched |
| Spirochaeta_2 | 8.5 | 0.8 | 0.0 | Group1_Enriched |
| Ramlibacter | 9.1 | 1.6 | 0.0 | Group1_Enriched |
| 1921_2 | 19.0 | 6.4 | 5.7 | Group1_Enriched |
| Actinomadura | 15.1 | 4.8 | 1.6 | Group1_Enriched |
| ADurbBin063_1 | 44.9 | 17.4 | 12.8 | Group1_Enriched |
| Nitrospira | 173.2 | 63.0 | 131.2 | Group2_Depleted |
| Rosenbergiella | 3.5 | 73.2 | 3.2 | Group2_Enriched |
| Burkholderia | 677.2 | 1250.9 | 567.9 | Group2_Enriched |
| alphaI_cluster | 1.2 | 12.2 | 0.0 | Group2_Enriched |
| Stenotrophomonas | 2.7 | 11.0 | 1.5 | Group2_Enriched |
| Caulobacter | 29.0 | 63.3 | 27.2 | Group2_Enriched |
| Massilia | 134.4 | 20.9 | 0.0 | Group3_Depleted |
| Haliangium | 312.6 | 81.5 | 23.3 | Group3_Depleted |
| Pseudomonas | 100.0 | 34.2 | 5.8 | Group3_Depleted |
| Pajaroellobacter | 291.7 | 98.7 | 17.4 | Group3_Depleted |
| G12_WMSP1 | 26.9 | 4.9 | 0.0 | Group3_Depleted |
| AKIW659 | 24.7 | 5.6 | 0.0 | Group3_Depleted |
| Nitrococcus | 22.8 | 7.6 | 0.0 | Group3_Depleted |
| Aliidongia | 18.2 | 8.5 | 0.8 | Group3_Depleted |
| Escherichia_Shigella | 8.4 | 14.3 | 1.5 | Group3_Depleted |
| Bdellovibrio | 26.0 | 20.0 | 1.1 | Group3_Depleted |
| bacterium | 5.5 | 7.9 | 0.4 | Group3_Depleted |
| Gemmatimonas | 34.8 | 39.3 | 7.7 | Group3_Depleted |
| Asticcacaulis | 13.3 | 14.7 | 2.8 | Group3_Depleted |
| Actinospica | 428.6 | 461.1 | 882.0 | Group3_Enriched |
| Bacillus | 588.8 | 578.6 | 1129 | Group3_Enriched |
| Microbispora | 9.1 | 1.7 | 3.4 | nosig |
| Brevundimonas | 2.0 | 8.2 | 5.7 | nosig |
| Pedomicrobium | 6.0 | 1.1 | 1.8 | nosig |
| Ammoniphilus | 16.6 | 7.8 | 12.9 | nosig |
| Arcticibacter | 3.1 | 0.4 | 0.4 | nosig |
| Gemella | 5.4 | 1.3 | 0.9 | nosig |
| Elizabethkingia | 5.4 | 1.3 | 1.1 | nosig |
| Methylovirgula | 159.5 | 230.2 | 249.6 | nosig |
| Turicibacter | 4.9 | 1.3 | 1.4 | nosig |
| Rhodopila | 34.0 | 21.2 | 25.4 | nosig |
| Chloroflexi | 37.5 | 50.1 | 80.2 | nosig |
| Clostridium | 1.3 | 3.8 | 1.9 | nosig |
| Fonticella | 2.0 | 4.2 | 0.8 | nosig |
| Lactobacillus | 17.8 | 24.5 | 36.7 | nosig |
| Catenulispora | 338.1 | 403.1 | 447.8 | nosig |
| Acidisoma | 9.0 | 5.9 | 3.3 | nosig |
| Pedosphaera | 8.2 | 11.2 | 12.3 | nosig |
| Bordetella | 2.9 | 4.1 | 5.5 | nosig |
| Crossiella | 74.1 | 66.3 | 111.8 | nosig |
| Ellin6067 | 7.6 | 5.9 | 2.1 | nosig |
| Not_Assigned | 21531 | 23139 | 33612 | nosig |
| Clostridium | 1.9 | 2.6 | 2.7 | nosig |
| Ktedonobacter | 2.4 | 1.7 | 0.4 | nosig |
| Kitasatospora | 65.0 | 69.3 | 85.7 | nosig |
| Aneurinibacillus | 3.1 | 2.5 | 1.2 | nosig |
| Streptococcus | 3.2 | 2.5 | 0.4 | nosig |
| Candidatus_Solibacter | 492.4 | 509.1 | 650.9 | nosig |
| Rhodoplanes | 6.7 | 6.0 | 5.2 | nosig |
| Kineococcus | 1.5 | 1.3 | 1.8 | nosig |
| Sinomonas | 118.0 | 113.5 | 86.7 | nosig |
| Candidatus_Koribacter | 171.7 | 172.8 | 180.7 | nosig |
| Amycolatopsis | 28.4 | 28.4 | 38.5 | nosig |
|  | | | | |
